# Urinary Sodium Concentration Is an Independent Predictor of All-Cause and Cardiovascular Mortality in a Type 2 Diabetes Cohort Population

**DOI:** 10.1155/2017/5327352

**Published:** 2017-02-01

**Authors:** Pierre-Jean Saulnier, Elise Gand, Stéphanie Ragot, Lise Bankir, Xavier Piguel, Frédéric Fumeron, Vincent Rigalleau, Jean-Michel Halimi, Richard Marechaud, Ronan Roussel, Samy Hadjadj, SURDIAGENE Study group

**Affiliations:** ^1^CHU de Poitiers, Centre d'Investigation Clinique, Poitiers, France; ^2^Université de Poitiers, UFR Médecine Pharmacie, CIC1402, Poitiers, France; ^3^Inserm, CIC1402, Poitiers, France; ^4^CHU Poitiers, Pole DUNE, Poitiers, France; ^5^Inserm, UMRS1138, Centre de Recherche des Cordeliers, Paris, France; ^6^Université Paris 6 Pierre et Marie Curie, Centre de Recherche des Cordeliers, Paris, France; ^7^Université Paris 7 Denis Diderot, UMRS1138, Centre de Recherche des Cordeliers, Paris, France; ^8^CHU Bordeaux, Service d'Endocrinologie, Diabétologie, Maladies Métaboliques et Nutrition, Bordeaux, France; ^9^CHU Tours, Service de Néphrologie, Tours, France; ^10^Université de Tours, Faculté de Médecine, Tours, France; ^11^Inserm, U1082, Poitiers, France

## Abstract

*Objective.* Sodium intake is associated with cardiovascular outcomes. However, no study has specifically reported an association between cardiovascular mortality and urinary sodium concentration (U_Na_). We examined the association of U_Na_ with mortality in a cohort of type 2 diabetes (T2D) patients.* Methods*. Patients were followed for all-cause death and cardiovascular death. Baseline U_Na_ was measured from second morning spot urinary sample. We used Cox proportional hazard models to identify independent predictors of mortality. Improvement in prediction of mortality by the addition of U_Na_ to a model including known risk factors was assessed by the relative integrated discrimination improvement (rIDI) index.* Results*. Participants (*n* = 1,439) were followed for a median of 5.7 years, during which 254 cardiovascular deaths and 429 all-cause deaths were recorded. U_Na_ independently predicted all-cause and cardiovascular mortality. An increase of one standard deviation of U_Na_ was associated with a decrease of 21% of all-cause mortality and 22% of cardiovascular mortality. U_Na_ improved all-cause and cardiovascular mortality prediction beyond identified risk factors (rIDI = 2.8%, *P* = 0.04 and rIDI = 4.6%, *P* = 0.02, resp.).* Conclusions*. In T2D, U_Na_ was an independent predictor of mortality (low concentration is associated with increased risk) and improved modestly its prediction in addition to traditional risk factors.

## 1. Introduction 

Patients with diabetes mellitus, and particularly type 2 diabetes (T2D), represent a large and growing population at increased risk of cardiovascular events and mortality. Recent data have confirmed that patients with diabetes have approximately twice as high a risk of mortality and vascular diseases as individuals without diabetes [1]. Blood pressure has been recognized as a key contributor to increased cardiovascular mortality [2].

Recent large-scale data have shown that estimated sodium intake was positively related to blood pressure [3]. Interestingly, in the general population but also in high cardiovascular risk, epidemiological data suggest a J-shape curve relationship between sodium intake and cardiovascular outcomes [4]. In most of these studies, sodium intake was indirectly assessed from sodium urinary concentration. As hyperglycemia might lead to polyuria and then modify urinary solute concentrations, the relationship between sodium excretion and cardiovascular mortality needs to be specifically established in diabetes patients. One study on type 1 diabetes (T1D) [5] together with one work we recently reported in patients with T2D [6] suggested a nonlinear relationship between sodium excretion and all-cause death. In addition, it has been reported that low measured 24-hour sodium excretion was associated with increased mortality in patients with T2D [7]. However, until now no study has examined the association of urinary sodium concentration (U_Na_) and mortality in T2D patients. The measurement of spot U_Na_ is a simple method for epidemiological approach and can easily be used in clinical practice. For this reason, our aim was to assess the relationship between spot urine U_Na_ and mortality with an observational approach in a single center prospective cohort of T2D patients.

## 2. Materials and Methods

### 2.1. Study Protocols and Participants

The present analyses include the subjects recruited in the SURDIAGENE study, a French single center inception cohort of T2D patients regularly visiting the Diabetes Department at Poitiers University Hospital, France [8]. Patients were included from 2002 to 2012 and outcome update has been performed every 2 years since 2007. The Poitiers University Hospital Ethics Committee approved the design (CPP Ouest III). All participants in the study gave their informed written consent.

### 2.2. Definition of Clinical Outcomes

The primary outcome was the occurrence of all-cause death. Hospital records, interviews with general practitioners, and the French death registry were used by an independent adjudication committee to establish death and its cause. The secondary outcome was the occurrence of cardiovascular death, defined according to the International Classification of Diseases, Tenth Revision (ICD-10, codes I00-I99). Vital status of all study participants was confirmed through December 31, 2013.

### 2.3. Procedures

Blood samples and second morning urine samples were obtained in patients after an overnight fast. Seated blood pressure was measured twice at 3 min intervals using a semiautomated device (Dinamap1846, Critikon, Tampa, FL, USA) after 10 min of seated rest. Systolic blood pressure (SBP) and diastolic blood pressure (DBP) levels were then calculated from the mean of the 2 consecutive measures.

### 2.4. Laboratory Methods

Serum and urine creatinine and urinary albumin concentrations were measured by nephelometry on a Modular System P (Roche Diagnostics GmbH, Mannheim, Germany). Renal function was estimated by glomerular filtration rate (eGFR) using the Chronic Kidney Disease Epidemiology formula [9]. Glycated hemoglobin was determined using a high performance liquid chromatography method with a ADAMS A1C HA-8160 analyzer (Menarini, Florence, Italy). Urinary sodium and potassium concentrations (U_K_) were determined by indirect potentiometry. Estimated daily sodium intake was derived from U_Na_ measured on spot urine according to Kawasaki et al.'s [10] and to Tanaka et al.'s formulas [11]. This approach has been shown to provide reliable information in large epidemiological studies [12]. The urine to plasma creatinine ratio (U : P_creat_) was calculated as an estimate of the overall urine concentration [13]. Glycosuria was semiquantitatively assessed using a urine dipstick Multistix 8SG (Siemens Healthcare Diagnostics Ltd, Camberley, UK). Plasma N-terminal of pro-brain natriuretic peptide (NT-proBNP) and copeptin concentrations (a surrogate for vasopressin) were measured in fasting plasma-EDTA samples, collected at baseline, and kept frozen at −80°C at Poitiers Biological Resources Center (BB-0033-00068). Plasma concentrations of NT-proBNP were measured using an electrochemiluminescent immunoassay (Elecsys proBNP II assay, Roche Diagnostics GmbH, Mannheim, Germany). An automated immunofluorescent sandwich immunoassay was used (BRAHMS Copeptin US KRYPTOR CT-proAVP; Thermofisher Scientific, Hennigsdorf, Germany) for the copeptin measurement.

### 2.5. Statistical Analysis

Patient characteristics are expressed as means ± standard deviation (SD) or medians (interquartile range) for skewed distributions. Groups were compared using the *χ*^2^ test for categorical variables or parametric (ANOVA) or nonparametric (Kruskal-Wallis) tests for continuous variables. Skewed variables were log-transformed in order to normalize their distribution. Associations between U_Na_ and other variables were tested with Spearman's rank correlation test or linear regression analysis where estimates were standardized by calculating them for 1-SD increment of each continuous variable. To model the association between estimated sodium excretion and clinical outcomes, we used restricted cubic splines. Based on restricted cubic spline analysis for sodium excretion and cardiovascular death, we selected 5–6.99 g/day as the reference category because this was the range associated with the lowest risk. The time to event was plotted according to the tertiles of U_Na_, U_K_, and U : P_creat_ with cumulative incidence curves, and comparison was made using the Log-rank test. We estimated the risk of outcome associated with U_Na_ using a Cox proportional hazard model. The effect of interaction of some variables on the relationship between U_Na_ and survival was examined by adding a corresponding interaction term in the model. Hazard ratios (HR) were standardized by calculating them for 1-SD increment of each continuous variable. A univariate model and two multivariate models were considered. The first multivariate model (Model A) was adjusted for age, sex, U_Na_, U_K_, U : P_creat_, and estimated 24 h Na excretion. Manual backward selection procedure was applied to identify the best fitted Cox proportional hazard model for independent variables in the prediction of all-cause and cardiovascular mortality. Variables associated with U_Na_ were all selected and then removed from the model when *P* value ≥ 0.05 until all the remaining variables were significant. Generalized *c*-statistics [14] were calculated for models that included or excluded U_Na_ to the best fitted model and comparisons between nested models were performed by likelihood ratios tests. The relative integrated discrimination improvement (rIDI) index was calculated to assess the improvement in 5.7-year mortality risk prediction for U_Na_ in addition to covariates of the best fitted model [15]. The 5.7-year risk was selected as it corresponds to the median of follow-up.

Three sensitivity analyses were performed. Firstly, patients with previous severe CKD, defined as eGFR < 30 mL·min^−1^ · 1.73 m^−2^ or history of renal replacement therapy at baseline, were omitted. Secondly, to test whether low U_Na_ or low SBP reflects chronic heart failure (CHF), we excluded patients with cardiovascular history (defined as prior myocardial infarction or stroke) at baseline. As we did not have information on CHF per se, history of cardiovascular disease was used as a surrogate for CHF. Thirdly, we used the competing risk model to estimate the subdistribution HR for cardiovascular death, while accounting for the competing risk of noncardiovascular deaths according to Fine and Gray method [16].* P* values < 0.05 were considered statistically significant. Statistical analyses were performed with SAS version 9.3 (SAS Institute, Cary, NC).

## 3. Results

### 3.1. Baseline Characteristics

The population studied included 1,439 patients with follow-up data. The mean value of U_Na_ was 89 ± 41 mmol/L. The mean value of 24-hour Na excretion was 4.6 ± 1.8 g/day. The clinical and biological characteristics of the patients are presented according to baseline U_Na_ tertiles (Table 1). Interestingly, people in the low U_Na_ tertile had a lower SBP and DBP compared to those in the intermediate or high U_Na_ tertiles (*P* = 0.012 and *P* < 0.001, resp.).

Urinary sodium concentration was associated with age, sex, history of cardiovascular disease, use of diuretics, use of insulin, eGFR, uACR, U_K_, U : P_creat_, estimated 24 h Na excretion, NT-proBNP, and glycosuria (Table 2). Patients with glycosuria had significantly higher U_Na_ compared to nonglycosuric patients (92.2 ± 39.0 versus 86.7 ± 42.0 mmol/L; *P* = 0.016).

### 3.2. All-Cause Mortality during Follow-Up

**Figure 1:**
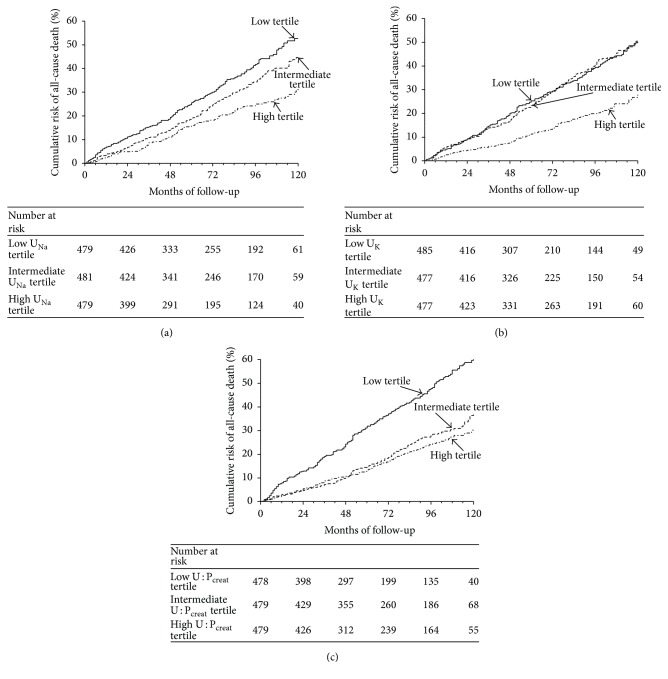
Cumulative risk of all-cause death according to urinary solute tertiles. (a) Kaplan-Meier survival curves are displayed according to tertiles of urinary sodium concentration (U_Na_) at baseline. Patient grouping: solid line indicates high U_Na_ tertile patients; dashed line indicates intermediate U_Na_ tertile patients; dotted line indicates low U_Na_ tertile patients. *P* value Log-rank test < 0.001. (b) Kaplan-Meier survival curves are displayed according to tertiles of urinary potassium concentrations (U_K_) at baseline. Patient grouping: solid line indicates high U_K_ tertile patients; dashed line indicates intermediate U_K_ tertile patients; dotted line indicates low U_K_ tertile patients. *P* value Log-rank test < 0.001. (c) Kaplan-Meier survival curves are displayed according to tertiles of urine to plasma creatinine concentration ratio (U : P_creat_) at baseline. Patient grouping: solid line indicates high U : P_creat_ tertile patients; dashed line indicates intermediate U : P_creat_ tertile patients; dotted line indicates low U : P_creat_ tertile patients. *P* value Log-rank test < 0.001.

The median follow-up was 5.7 years (interquartile range 3.1–8.8 years), during which time 429 all-cause deaths (incidence rate 51 per 1000 person-years [95% CI, 46–55]) and 254 cardiovascular deaths occurred (incidence rate 30 per 1000 person-years [95% CI, 26–33]). The causes of noncardiovascular death were adjudicated as follows: cancer (*n* = 58), infection (*n* = 41), and other causes (*n* = 76). When patients were classified according to the U_Na_ tertile distribution, those in the low U_Na_ tertile had the highest cardiovascular mortality (Log-rank *P* < 0.0001) as illustrated in Figure 1(a). Interestingly, a similar relationship was found between all-cause mortality and U_K_ (Figure 1(b)) and U : P_creat_ (Figure 1(c)), and even the relationship was graphically less gradual. Urinary sodium concentration, but not U : P_creat_, was a predictor of all-cause death after adjustment for U_K_, U : P_creat_, and estimation of 24 h urinary sodium excretion (Table 3). Urinary sodium concentration remained an independent predictor of all-cause death in the final best fitted model: age, gender, use of insulin, uACR, copeptin, and NT-proBNP (Table 4). After adjustments for covariates of this best fitted model all-cause mortality was 21% lower for every 1-SD increase of U_Na_ (*P* < 0.001). The addition of U_Na_ increased the *c*-statistic from 0.776 to 0.780 (difference in *c*-statistic = 0.004, *P* < 0.001; rIDI = 2.8%,* P* = 0.04).

### 3.3. Cardiovascular Mortality during Follow-Up

**Figure 2:**
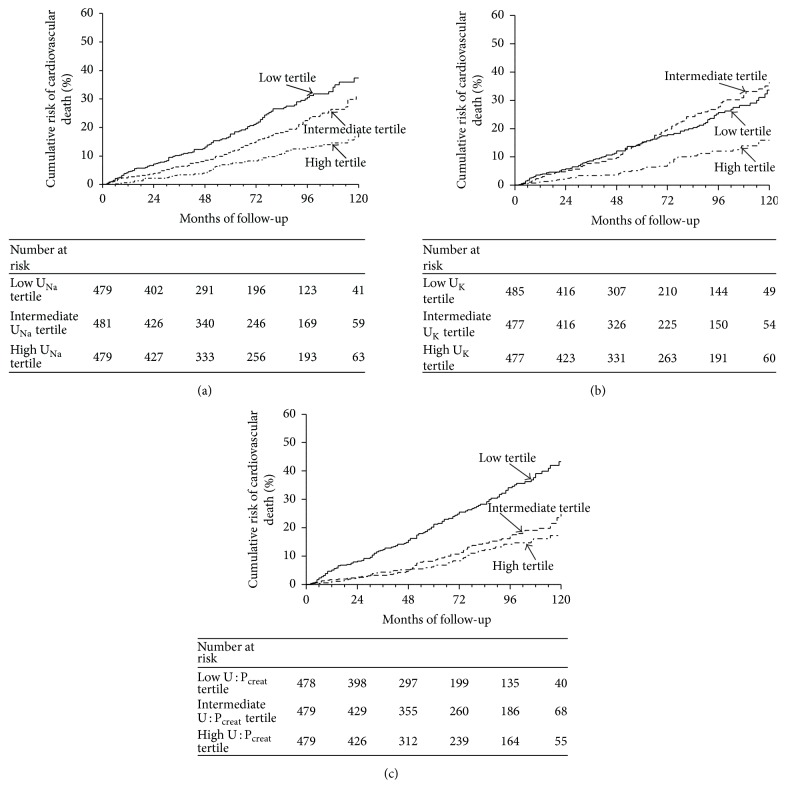
Cumulative risk of cardiovascular death according to urinary solute tertiles. (a) Kaplan-Meier survival curves are displayed according to tertiles of urinary sodium concentration (U_Na_) at baseline. Patient grouping: solid line indicates high U_Na_ tertile patients; dashed line indicates intermediate U_Na_ tertile patients; grey line indicates low U_Na_ tertile patients.* P* value Log-rank test < 0.001. (b) Kaplan-Meier survival curves are displayed according tertiles of urinary potassium concentrations (U_K_) at baseline. Patient grouping: solid line indicates high U_K_ tertile patients; dashed line indicates intermediate U_K_ tertile patients; dotted line indicates low U_K_ tertile patients. *P* value Log-rank test < 0.001. (c) Kaplan-Meier survival curves are displayed according to tertiles of urine to plasma creatinine concentration ratio (U : P_creat_) at baseline. Patient grouping: solid line indicates high U : P_creat_ tertile patients; dashed line indicates intermediate U : P_creat_ tertile patients; dotted line indicates low U : P_creat_ tertile patients. *P* value Log-rank test < 0.001.

We found a similar pattern and a significant relationship between cardiovascular mortality and tertiles of U_Na_ (Figure 2(a)), U_K_ (Figure 2(b)), and U : P_creat_ (Figure 2(c)). Urinary sodium concentration, but not U : P_creat_, was a also predictor of cardiovascular death after adjustment for U_K_, U : P_creat_, and estimation of 24 h urinary sodium excretion (Table 5).

In the best fitted model for cardiovascular death prediction (age, sex, BMI, use of insulin, uACR, U_Na_, and NT-proBNP), U_Na_ was also an independent risk factor for cardiovascular death. In this model, cardiovascular mortality was 24% lower for every 1-SD increase of U_Na_ (*P* < 0.0001) as detailed in Table 6.

The addition of U_Na_ increased the *c*-statistic from 0.803 to 0.807 (difference in *c*-statistic = 0.004, *P* = 0.0002; rIDI = 4.6%, *P* = 0.02). There was no significant difference of all-cause and cardiovascular death according to glycosuria (Log-rank *P* = 0.80 and 0.60, resp.). We found no interaction of glycosuria, use of diuretics, or use of insulin with the association between urinary concentrations and either all-cause or cardiovascular death. 24-hour Na excretion considered as a 3-category variable (reference group = 5–5.99 g/day) was not significantly associated with all-cause or cardiovascular mortality; even graphical representation of the relationship evocated nonlinear J-shape relationship. Considering Tanaka's formulas instead of Kawasaki's for estimating 24-hour Na excretion did not modify the results of the final Cox models.

Because severe CKD is an established independent risk factor for cardiovascular complications, we focused on a subset of patients with eGFR ≥ 30 mL·min^−1^ · 1.73 m^−2^ and no history of renal replacement therapy (*n* = 1,035 patients). In this subgroup, U_Na_ was not correlated with SBP (*r* = 0.04, *P* = 0.24). All-cause and cardiovascular deaths were registered in 229 (22%) and 123 (12%) patients, respectively. Results were unchanged with a clear graded protective effect of U_Na_: all-cause and cardiovascular mortality were 19% and 21% lower for every 1-SD U_Na_ increase in the corresponding best fitted model; *P* = 0.005 and *P* = 0.02, respectively.

In the group of patients without any personal cardiovascular history at baseline (*n* = 1,151 patients), all-cause death and cardiovascular death were registered in 304 (26%) and 165 (14%) patients, respectively. Results were similar as U_Na_ remained significant predictor of mortality (all-cause mortality and cardiovascular mortality were 18% and 23% lower for every 1-SD increase of U_Na_ in the corresponding best fitted model; *P* = 0.003 and *P* = 0.005, resp.). Accounting for the competing risk of noncardiovascular mortality in a Fine and Gray analysis (Table 6) did not modify results and U_Na_ remained independently associated with a decrease in risk of cardiovascular death (*P* = 0.007).

Presence of glycosuria or use of diuretics/insulin did not interact with the association of U_Na_ and the risk of mortality, whatever its cause, in these subpopulations.

## 4. Discussion

The present study reveals a significant negative prognostic value of U_Na_ for all-cause and cardiovascular mortality in a prospective cohort of T2D patients. Even though they had a lower blood pressure at baseline, patients in the low U_Na_ tertile had a significantly increased risk of all-cause and cardiovascular deaths. These association appeared to be independent from other known cardiovascular risk factors and also from U_K_, estimated daily sodium intake, index of urine concentration (U : P_creat_), and glycosuria. Nevertheless, the extent to which U_Na_ improves risk prediction is modest according to the rIDI and the change in *c*-statistics and its value in clinical practice remain to be determined.

Data from prospective epidemiologic studies focusing on estimated or measured 24 h urinary sodium excretion identified inconsistent association with cardiovascular complications [17–20]. Interestingly estimation of 24 h urinary sodium excretion in this work presented a magnitude in accordance with other reports [21, 22]. However recent T1D and large-scale general population studies have identified a J-shape relationship [5, 23]. We also reported, in the T2D SURDIAGENE study population, this nonlinear pattern [6]. A recent publication showed that serum sodium concentration was associated with mortality independently of copeptin in a distinct general practitioner-recruited T2D population [24].

Nevertheless clinical trials have demonstrated that reduced sodium intake lowered blood pressure [25]. However interventions lowering salt intake have not established a definite clinical benefit either in the general or more particularly in the T2D population [26–28]. The low Na excretion may be due to insulin therapy, which is more frequent in the low tertile of U_Na_. The antinatriuretic effect of insulin may contribute to the relationship between insulin resistance and hypertension [29], and patients in the low U_Na_ tertile also require less antihypertensive therapy. The question whether low U_Na_ may play a role in higher mortality in insulin-treated patients as recently reported [30] will need more research.

Our key finding is that U_Na_ constitutes a prognostic factor for all-cause and cardiovascular mortality in T2D patients. To the best of our knowledge, no similar conclusions had previously been reached. Interestingly, our data support the hypothesis that the kidney capacity to excrete water and solute in the urine is implicated in the occurrence of fatal cardiovascular complications. This hypothesis would benefit from reanalyzing previous studies. However, our current findings propose a shift of paradigm from simple sodium intake to a more complex phenomenon associated with cardiovascular death: the kidney's ability to excrete sodium. As sodium concentration is lower in urine compared to plasma, the kidney independently regulates the amount of water required to excrete solutes. Our data on U_Na_ but also on U : P_creat_ support the likelihood that a higher excretion of water leading to a lower U_Na_ is particularly ominous. Though urinary osmolarity is lacking, this speculation needs validation.

The pathophysiology underlying such relationships remains unclear. The potentially involved biological systems are those contributing to the regulation of sodium and volemia. The natriuretic peptides and vasopressin, the renin-angiotensin-aldosterone system (RAS), or the sympathetic nervous system (SNS) could all play a role.

Low U_Na_ was associated with low SBP levels in our population. Because these conditions could be present in CHF, we adjusted our analyses for NT-proBNP, a marker of CHF, and we analyzed separately patients without history of cardiovascular disease. Indeed Na and water excretion was impaired in CHF [31] whereas NT-proBNP is markedly increased. Nevertheless, adjustment for NT-proBNP did not attenuate the prognostic value of U_Na_. We also tried to determine whether low U_Na_ could be related to arginine vasopressin (AVP), the hormone fine-tuning water excretion in the kidney collecting duct. Because AVP is difficult to measure, measurement of copeptin, the N-terminal part of the AVP prohormone, is now widely used as a proxy of AVP concentration [32]. High copeptin concentration was shown to be an important biomarker for renal and all-cause mortality in 2 independent cohorts of T2D patients [24, 33]. As copeptin is secreted when water reabsorption is required, its concentration was expected to be higher in patients with high U_Na_. Indeed the higher U_Na_, the higher copeptin concentration. In addition, as glycosuria may contribute to fluid loss in T2D patients, we confirmed that plasma copeptin was higher in patients with glycosuria compared to nonglycosuric patients (data not shown). As the deleterious effect of low U_Na_ remained significant after adjustment for copeptin concentration, plasma copeptin concentration is unlikely to be the sole contributing factor between U_Na_ and cardiovascular death.

We then examined the possible implications of RAS activation. This hormonal pathway was shown to be associated with several cardiovascular and renal outcomes following genetic and pharmacologic approaches [34]. The RAS is activated when sodium intake decreases, as has recently been shown in patients with diabetes [35]. Even if the RAS could be a possible explanation for our findings, two factors speak against a deleterious role of the RAS. First, our observations were barely affected when adjusting for RAS blockade treatment into multivariate models. Second, the activation of the RAS leads to reduced urinary sodium excretion at the expense of increased potassium excretion. As sodium and potassium move in opposite directions when RAS is activated, our finding of an increased risk for cardiovascular death with both low sodium and low potassium suggests that RAS activation is not the main contributor.

A third explanation focused on renal SNS. Diabetes is associated with autonomic dysfunction and sympathetic/parasympathetic imbalance [36]. Overactivity of the renal SNS leads to increase in renin secretion rate, decrease in urinary sodium excretion (by increasing renal tubular sodium reabsorption), and decrease in renal blood flow [37]. In addition, decreased sodium intake leads to an increase in catecholamines [34]. Our observations were barely affected when adjusting for beta blocker treatment into multivariate models. Direct and accurate measurement of the autonomic nervous system was not available at baseline. Even though it has yet to be comprehensively detailed, its role could still help to explain our results. While possible use of low U_Na_ as a valuable surrogate for overactivity of renal SNS requires future confirmation, it can be speculated that early detected SNS dysregulation may reveal possible targets for pharmacological intervention (such as beta blockers), but also for nonpharmacological interventions such as renal denervation or barostimulation that are currently under evaluation [38].

Some limitations in our study must be acknowledged. This study was not designed to collect 24-hour urine and exploration of the relation between U_Na_ and measured daily urinary sodium excretion in our cohort was therefore beyond our scope. We based our analysis on a single voided morning urine sample, which represents usual individual solute [39]. Misclassification of patients due to fluctuations of solute excretion should rather lead to false negative, rather than false positive findings.

While urinary sodium excretion rate is known to exhibit a daily (circadian) to weekly (circaseptan) pattern [40, 41], in the present study, one-time spot urinary samples were drawn at close times for all patients. However 24-hour urine samples cannot evidence impaired circadian pattern of water and Na excretion rates in diabetes, if any, as shown in the metabolic syndrome [42].

We performed no dietary assessment of salt or fluid intake, and information regarding timed urine volume was not available. Although 24-hour urine samples are theoretically more satisfactory as a surrogate for daily sodium excretion [12], they present some pitfalls in this type of study. The collection of urine may not be complete, and incompleteness may be systematically biased, in which case, statistical association may be misleading. Our study is based on a single morning urine sample, similar to what has been considered in other recent studies [43]. Even if multiple-time spots mean U_Na_ may provide less intrasubject variability compared to one-time spot U_Na_, it is unlikely to affect intersubject variability. In addition, this approach of repeated urinary sampling seems not feasible for large epidemiologic studies. However, our study also has a number of strengths. The size of our cohort allows detection of low level associations. An independent adjudication committee validated all single events. Three independently measured urinary variables (U_Na_, U_K_, and U : P_creat_) provided similar trends.

## 5. Conclusions

In conclusion, we have evidenced that U_Na_ measured in the morning can be proposed as new a biomarker of risk of all-cause and cardiovascular mortality, in type 2 diabetes patient.

## Figures and Tables

**Table 1:** Baseline characteristics according to tertiles of urinary sodium concentration.

	All (*n *= 1,439)	Tertiles of urinary sodium concentration			*P* value
		Low	Intermediate	High	
		<69 mmol/L (*n* = 479)	69–103 mmol/L (*n *= 481)	>103 mmol/L (*n* = 479)	
Clinical variables					
Age (year)	65.3 ± 10.7	66.1 ± 10.6	65.6 ± 10.5	64.0 ± 10.8	0.008
Male, *n* (%)	835 (58%)	252 (53%)	282 (59%)	301 (63%)	0.006
Body mass index (kg/m^2^)	31.2 ± 6.3	31.2 ± 6.4	30.7 ± 6.0	31.9 ± 6.3	0.02
Active smoking, *n* (%)	152 (11%)	48 (10%)	48 (10%)	56 (12%)	0.66
Diabetes duration (years)	14.5 ± 10.0	15.9 ± 10.1	15.1 ± 10.4	12.5 ± 9.3	<0.001
HbA_1c_ (%)	7.8 ± 1.6	7.7 ± 1.7	7.8 ± 1.7	7.7 ± 1.5	0.47
History of cardiovascular disease, *n* (%)	276 (19%)	113 (24%)	87 (18%)	76 (16%)	0.005
Systolic blood pressure (mmHg)	132 ± 18	131 ± 18	134 ± 18	133 ± 16	0.03
Diastolic blood pressure (mmHg)	72 ± 11	71 ± 11	73 ± 10	73 ± 12	0.002
Medications use					
Any Diabetes therapy, *n* (%)	1,378 (96%)	462 (97%)	457 (95%)	459 (96%)	0.62
Oral antidiabetic agent, *n* (%)	910 (63%)	279 (58%)	294 (61%)	337 (71%)	<0.001
Insulin, *n* (%)	864 (60%)	322 (67%)	292 (61%)	250 (52%)	<0.001
Any antihypertensive therapy, *n* (%)	1189 (83%)	426 (89%)	394 (82%)	369 (77%)	<0.001
Diuretics, *n* (%)	655 (46%)	268 (57%)	210 (44%)	177 (37%)	<0.001
RAAS blockers, *n* (%)	900 (63%)	316 (66%)	301 (63%)	283 (60%)	0.05
Beta blockers, *n* (%)	483 (34%)	185 (39%)	163 (34%)	135 (28%)	0.002
Calcium channel blockers, *n* (%)	448 (31%)	162 (34%)	146 (31%)	140 (29%)	0.23
Renal parameters					
eGFR (ml·min^−1^ per 1.73 m^−2^)	73 ± 25	65 ± 27	73 ± 24	81 ± 19	<0.001
uACR (mg/g)	3.0 (12.9)	4.1 (18.2)	3.5 (15.1)	2.4 (7.6)	<0.001
Diabetic nephropathy (normo-/micro-/macroalbuminuric): *n* (%)	528 (43%)/421 (34%)/293 (24%)	163 (40%)/119 (29%)/127 (31%)	173 (41%)/149 (35%)/100 (24%)	192 (47%)/153 (37%)/66 (16%)	<0.001
U_Na_ (mmol/L)	89 ± 41	45 ± 15	85 ± 10	135 ± 26	<0.001
U_K_ (mmol/L)	52 ± 25	47 ± 26	51 ± 24	59 ± 24	<0.001
U : P_creat_	91 (89)	70 (85)	82 (78)	114 (84)	<0.001
Estimated 24 h Na excretion (g/day)	4.6 ± 1.8	3.5 ± 1.4	4.9 ± 1.5	5.5 ± 1.7	
NT-proBNP (pg/mL)	113 (270)	155 (530)	113 (241)	83 (147)	
Copeptin (pmol/L)	6.7 (8.0)	6.7 (10.7)	6.2 (7.2)	7.3 (6.7)	0.07
Presence of glycosuria: *n* (%)	494 (34%)	143 (30%)	170 (35%)	181 (38%)	0.03

Quantitative variables are described by mean ± SD or median (IQR).

History of cardiovascular disease = history of stroke or myocardial infarction; RAAS blockers = angiotensin receptor blockers and/or ACE inhibitors. eGFR, CKD EPI equation; uACR, urinary albumin/creatinine ratio; U_Na_, urinary sodium concentration; U_K_, urinary potassium concentration; U : P_creat_, urinary to plasma creatinine concentration ratio; NT-proBNP, N-terminal pro-brain natriuretic peptide.

**Table 2:** Association of clinical and biological characteristic variables with measured urinary sodium concentration (Spearman's correlation).

	Estimate (95% CI)	*P* value
Age	−0.09 (−0.15 to −0.04)	<0.001
Male gender	−0.16 (−0.26 to −0.05)	0.004
History of cardiovascular disease	−0.18 (−0.31 to −0.05)	0.008
Use of diuretics	−0.30 (−0.41 to −0.20)	<0.001
Use of insulin	−0.24 (−0.34 to −0.14)	<0.001
eGFR	0.28 (0.23 to 0.33)	<0.001
uACR	−0.14 (−0.19 to −0.09)	<0.001
U_K_	0.18 (0.13 to 0.24)	<0.001
U : P_creat_	0.18 (0.13 to 0.23)	<0.001
estimated 24 h Na excretion	0.29 (0.26 to 0.31)	<0.001
NT-proBNP	−0.22 (−0.27 to −0.17)	<0.001
Glycosuria	0.13 (0.03 to 0.24)	0.02

Estimates are calculated for 1-SD increment of continuous variables.

eGFR, estimated glomerular filtration rate; uACR, urinary albumin/creatinine ratio; U_K_, urinary potassium concentration; U : P_creat_, urine to plasma creatinine ratio; NT-proBNP, N-terminal pro-brain natriuretic peptide.

uACR, copeptin, and NT-proBNP are log-transformed.

**Table 3:** Cox proportional hazards models for the risk of all-cause death.

	Univariate	*P* value	Model A	*P* value
	Crude hazard ratio (95% CI)		Adjusted hazard ratio (95% CI)	
U_Na_	0.71 (0.64 to 0.79)	<0.001	0.73 (0.63 to 0.85)	<0.001
U : P_creat_	0.61 (0.54 to 0.70)	<0.001	0.99 (0.84 to 1.16)	0.86

Hazard ratios were standardized by calculating them for 1-SD increment of each continuous variable.

Model A = U_Na_, U_K_, U : P_creat_, age, sex and estimated 24 h-Na excretion.

**Table 4:** Multivariate Cox proportional hazards model for the risk of all-cause death (best fitted model).

	Adjusted hazard ratio (95% CI)	*P* value
Age	1.78 (1.58 to 2.01)	<0.001
Sex: (reference = men)	0.68 (0.55 to 0.84)	<0.001
Use of Insulin	1.51 (1.21 to 1.89)	<0.001
eGFR	1.16 (1.02 to 1.32)	0.03
uACR	1.30 (1.17 to 1.44)	<0.001
U_Na_	0.79 (0.71 to 0.88)	<0.001
Copeptin	1.19 (1.06 to 1.33)	0.004
NT-proBNP	1.56 (1.38 to 1.75)	<0.001

Hazard ratios were standardized by calculating them for 1-SD increment of each continuous variable.

eGFR, estimated glomerular filtration rate; uACR, urinary albumin to creatinine concentration ratio; U_Na_, urinary sodium concentration; NT-proBNP, N-terminal pro-brain natriuretic peptide.

uACR, copeptin, and NT-proBNP are log-transformed.

**Table 5:** Cox proportional hazards models for the risk of cardiovascular death.

	Univariate	*P* value	Model A	*P* value
	Crude hazard ratio (95% CI)		Adjusted hazard ratio (95% CI)	
U_Na_	0.65 (0.56 to 0.74)	<0.001	0.66 (0.54 to 0.81)	<0.001
U : P_creat_	0.56 (0.47 to 0.67)	<0.001	0.89 (0.74 to 1.07)	0.33

Hazard ratios were standardized by calculating them for 1-SD increment of each continuous variable.

Model A = U_Na_, U_K_, U : P_creat_, age, sex and estimated 24 h-Na excretion.

**Table 6:** Risk of cardiovascular death according to Cox model [left] and Fine and Gray competing risk [right] model.

	Adjusted hazard ratio (95% CI)	*P* value	Adjusted subhazard ratio^*∗*^ (95% CI)	*P* value
Age	1.76 (1.50 to 2.07)	<0.001	1.53 (1.30 to 1.79)	<0.001
Sex: (reference men)	0.66 (0.50 to 0.86)	0.002	0.73 (0.56 to 0.95)	0.02
BMI	1.19 (1.04 to 1.36)	0.01	1.13 (0.99 to 1.28)	0.07
Use of Insulin	1.62 (1.20 to 2.19)	0.002	1.57 (1.15 to 2.14)	0.005
uACR	1.26 (1.11 to 1.42)	<0.001	1.19 (1.04 to 1.36)	0.01
U_Na_	0.76 (0.66 to 0.88)	<0.001	0.82 (0.71 to 0.95)	0.007
NT-proBNP	1.90 (1.66 to 2.18)	<0.001	1.80 (1.56 to 2.08)	<0.001

Hazard ratios were standardized by calculating them for 1-SD increment of each continuous variable. History of uACR, urinary albumin to creatinine concentration ratio; U_Na_, urinary sodium concentration; NT-proBNP, N-terminal pro-brain natriuretic peptide.

uACR and NT-proBNP are log-transformed.

^*∗*^Cox proportional subhazards ratios for the risk of cardiovascular death are computed according to Fine and Gray competing risk models when taking into account the competing risk of noncardiovascular death.
